# Multilevel challenges to engagement in HIV care after prison release: a theory-informed qualitative study comparing prisoners’ perspectives before and after community reentry

**DOI:** 10.1186/1471-2458-14-1253

**Published:** 2014-12-09

**Authors:** Danielle F Haley, Carol E Golin, Claire E Farel, David A Wohl, Anna M Scheyett, Jenna J Garrett, David L Rosen, Sharon D Parker

**Affiliations:** Department of Behavioral Sciences and Health Education, Rollins School of Public Health, 1518 Clifton Rd., NE, Atlanta, GA 30322 USA; Department of Health Behavior, Gillings School of Global Public Health, University of North Carolina, Chapel Hill, NC USA; Department of Medicine, School of Medicine, University of North Carolina, Chapel Hill, NC USA; Institute for Global Health and Infectious Diseases, University of North Carolina, 130 Mason Farm Road, Campus Box 7215, Chapel Hill, NC 27599 USA; College of Social Work, University of South Carolina, Columbia, SC 29208 USA; Latin America Program, Planned Parenthood Global, Miami, FL 33131 USA; The Miriam Hospital Division of Infectious Diseases and Brown University School of Medicine, Providence, RI 02906 USA; Cecil G. Sheps Center for Health Services Research, 725 Airport Road, Suite 305D, Campus Box 7110, Chapel Hill, NC 27599 USA

**Keywords:** HIV/AIDS, Incarceration, Social cognitive theory, Stigma, Qualitative research, Substance misuse

## Abstract

**Background:**

Although prison provides the opportunity for HIV diagnosis and access to in-prison care, following release, many HIV-infected inmates experience clinical setbacks, including nonadherence to antiretrovirals, elevations in viral load, and HIV disease progression. HIV-infected former inmates face numerous barriers to successful community reentry and to accessing healthcare. However, little is known about the outcome expectations of HIV-infected inmates for release, how their post-release lives align with pre-release expectations, and how these processes influence engagement in HIV care following release from prison.

**Methods:**

We conducted semi-structured interviews (24 pre- and 13 post-release) with HIV-infected inmates enrolled in a randomized controlled trial of a case management intervention to enhance post-release linkage to care. Two researchers independently coded data using a common codebook. Intercoder reliability was strong (kappa = 0.86). We analyzed data using Grounded Theory methodology and Applied Thematic Analysis. We collected and compared baseline sociodemographic and behavioral characteristics of all cohort participants who did and did not participate in the qualitative interviews using Fisher’s Exact Tests for categorical measures and Wilcoxon rank-sum tests for continuous measures.

**Results:**

Most participants were heterosexual, middle-aged, single, African American men and women with histories of substance use. Substudy participants were more likely to anticipate living with family/friends and needing income assistance post-release. Most were taking antiretrovirals prior to release and anticipated needing help securing health benefits and medications post-release. Before release, most participants felt confident they would be able to manage their HIV. However, upon release, many experienced intermittent or prolonged periods of antiretroviral nonadherence, largely due to substance use relapse or delays in care initiation. Substance use was precipitated by stressful life experiences, including stigma, and contact with drug-using social networks. As informed by the Social Cognitive Theory and HIV Stigma Framework, findings illustrate the reciprocal relationships among substance use, experiences of stigma, pre- and post-release environments, and skills needed to engage in HIV care.

**Conclusion:**

These findings underscore the need for comprehensive evidence-based interventions to prepare inmates to transition from incarceration to freedom, particularly those that strengthen linkage to HIV care and focus on realities of reentry, including stigma, meeting basic needs, preventing substance abuse, and identifying community resources.

## Background

A substantial proportion of HIV-infected men and women in the United States (US) are incarcerated [1–3], reflecting the endemicity and disproportionate burden of incarceration in communities most affected by HIV [4–6]. Although prison provides the opportunity for HIV diagnosis and access to in-prison care [7–10], following release from prison, many HIV-infected inmates experience clinical setbacks, including nonadherence to antiretroviral therapy (ART), elevations in viral load, and HIV disease progression [8, 10–14]. Given the high prevalence of HIV risk behaviors among HIV-infected former prison inmates (“releasees”) [15–17] and the increased risk of transmission associated with higher HIV viral loads [18], prison release represents a critical juncture in the health of the individual and often, his/her community. Following release, inmates struggle to prioritize engagement in HIV-related care in the face of poor access to medical care; limited health insurance, social benefits, and employment prospects; endemic poverty; and unstable housing [11, 13, 14, 19–24]. Underlying mental illness and substance use further complicate this transition [25].

Stigma serves as an additional barrier to accessing care for HIV-infected releasees [26–28] and experiences of HIV-related stigma have been associated with poor health outcomes among people living with HIV/AIDS (PLWHA) in the US [29–31]. The negative effect of stigma may be compounded for HIV-infected inmates, who also bear the stigma of incarceration and often substance use disorders, mental illness, and racial minority status [30]. HIV-infected inmates’ experiences of stigma [29–31] and its impact on engagement in HIV care can be informed by the HIV Stigma Framework (HSF), which explores how individuals experience stigma, and how these experiences affect psychological, health, and behavioral outcomes [32]. Using this framework, stigma is experienced by people living with HIV/AIDS (PLWHA) through at least three mechanisms: 1) Enacted stigma (the degree to which PLWHA believe they have experienced prejudice or discrimination); 2) Anticipated stigma (the degree to which PLWHA expect that they will experience prejudice or discrimination in the future); and 3) Internalized stigma (the degree to which PLWHA endorse the negative beliefs and feelings associated with HIV/AIDS about themselves) [32].

Although there is a rich literature documenting the challenges of HIV-infected prison inmates face upon release, less is known about how the expectations of HIV-infected prison inmates for engagement in HIV-related care and treatment prior to release compare to the realities of the environment they face upon community reentry, and how potential discrepancies between expectations and reality impact behaviors related to HIV care upon release. A richer understanding of how individual-level characteristics (e.g., substance use history) interact with the social and built environment (e.g., social networks) to influence HIV-related self-management behaviors and engagement in HIV care after prison release (e.g., linkage to care and adherence to ART) may inform the development of multilevel interventions designed to improve engagement in care in this vulnerable population. The Social Cognitive Theory (SCT) posits that individuals, their health behaviors (e.g., linkage to HIV care, adherence to HIV medication), and their physical and social environments are in constant interaction, modifying each other and shaping future health behaviors and associated health outcomes (reciprocal determinism) [33]. An individual’s behaviors may be further influenced by the value he or she places on certain outcomes (outcome expectations), and the individual’s confidence in his/her ability to perform (self-efficacy) and to regulate (self-regulation) these behaviors. The SCT has been successfully used to inform HIV management (e.g., improving ART adherence) in US populations [34–36] and can be applied to HIV management following release from prison. Inmates experience dramatic changes in their social and built environments following release; formal sanctions inherent in the correctional environment strongly promote abstinence from substance use and enforced adherence to antiretroviral medications (ARVs) and medical appointments. The impact of the environment on substance use disorders and engagement in HIV care comes into stark relief upon release from prison, when competing demands, such as the need for food and shelter, as well as the burdens of poverty, addiction, and changes in mental and physical health status may interfere with engagement in care for HIV.

This manuscript, informed by the SCT and HSF, explores qualitatively the expectations of HIV-infected prison inmates for engagement in HIV-related care and treatment as they prepare for release, the individual characteristics affecting their release experiences as they relate to engagement in care, the realities of the environment they face upon community reentry, and the confluent impact of these factors on their engagement in HIV care upon release.

## Methods

### Study design

**Figure 1:**
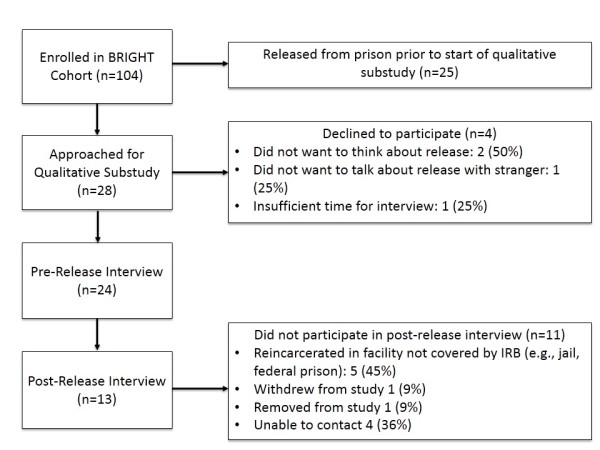
**Study flow diagram.**

We conducted semi-structured qualitative interviews with HIV-infected men and women before and after release from prison (Figure 1). We used a convenience sample of participants enrolled in the Bridges to Good Care and Treatment (BRIGHT) study (design previously described) [37], a randomized controlled trial testing the impact of strengths-based bridging case management (BCM) on post-release outcomes among HIV-infected individuals released from the North Carolina Department of Public Safety (NCDPS) Division of Adult Correction. BRIGHT eligibility criteria included: plans to return to one of twelve NC counties and being HIV-infected, within 12 to 4 weeks of projected release, ≥18 years old, and English-speaking. BRIGHT study participants were followed up to 48 weeks after release from prison. Participants in the control arm received NCDPS standard of care discharge planning prior to release only. Participants enrolled in the intervention arm received BCM up to six months following release from prison. Recruitment for the qualitative substudy was initiated approximately 8 months after the start of the parent study. Qualitative substudy eligibility criteria included: enrolled in parent study, pre-release, and willing/able to provide informed consent for the qualitative interview. Potential participants were approached after enrollment in the parent study, during a routine clinical appointment at the NCDPS infectious disease clinics. We approached 28 potential participants for the qualitative substudy during clinical appointments at the NCDPS infectious diseases clinics, of these, 24 (86%) agreed to participate. All study activities were approved by the Institutional Review Boards (IRB) at the University of North Carolina at Chapel Hill School of Medicine and the NCDPS, as well as the Office of Human Research Protection at the US Department of Health and Human Services. Each participant provided informed consent prior to study procedures. This manuscript adheres to RAT guidelines for reporting qualitative studies.

### Semi-structured interview guides

At pre-release, we assessed expectations for the release process and post-release life, the impact release would have on relationships with people both inside and outside of prison, and the role of HIV infection in post-release life. Post-release interviews explored participants’ living situations, social networks, experiences living with HIV, and how pre-release expectations compared to their lived experiences.

### Data collection

Interviews lasted 30 to 90 minutes and were recorded and transcribed. Pre-release interviews took place in a private room at a NCDPS correctional facility with only the participant and interviewer present. Enrollment for the qualitative substudy was stopped when saturation of themes was apparent based on real-time review of transcripts (i.e., no new themes emerged from the data). Post-release interviews with participants who completed pre-release interviews took place within one year of release in community settings that provided adequate privacy (e.g., library). Participants who were reincarcerated in NCDPS correctional facilities before completing the post-release interview were interviewed in a private room at the prison medical facility. Baseline quantitative demographic and behavioral data (e.g., substance use, unmet needs) were obtained at enrollment in the parent study through researcher-administrated questionnaires. Survey questions included standardized and modified scales or items [37]. Unmet needs were measured using HIV Cost and Services Utilization Study (HCSUS) instruments [38], substance use was assessed with a survey developed by the Enhancing Prevention with Positives Evaluation Center (EPPEC) [39]. Clinical markers (e.g., CD4 counts) were abstracted from prison medical records.

### Qualitative data analysis

We developed the codebook and conducted the data analysis using the principles of Grounded Theory methodology [40, 41] and Applied Thematic Analysis [42]. Two trained researchers independently coded interviews using a common codebook, first applying open codes for all interview statements. Codes were reviewed by the research team and integrated into major salient themes by identifying and exploring axial connections among codes, taking into consideration code frequencies. Emerging themes and resulting axial connections were then used to construct a theoretical framework, which was subsequently compared to existing health behavior theories to examine whether data-driven constructs reflected existing theoretical frameworks. Data were analyzed using Nivo9 qualitative data analysis software (QSR International Pty Ltd. Version 9, 2010).

### Intercoder reliability

We assessed intercoder reliability (ICR) using the NVivo9 ICR function early and midway through coding using five transcripts (three pre-release and two post-release). Codes with a kappa below 0.8 were discussed, a consensus reached, and the codebook was revised as needed. We then recoded all interviews based on the revised codebook. The final overall kappa was 0.86.

### Quantitative data analysis

To characterize the sample, we assessed medians and interquartile ranges for continuous variables and tabulated categorical variables using SAS version 9.3 (SAS Inc., Cary, NC). We compared baseline characteristics of: 1) the parent study participants who did not participate in the qualitative interviews (n = 81) and the qualitative subset (n = 23) and 2) the qualitative subset participants who completed the pre-release interview only (n = 10) versus the participants who completed both the pre- and post-release qualitative interviews (n = 13). We assessed demographics, incarceration history, alcohol and illicit substance use behaviors in the three months prior to incarceration, HIV diagnosis and treatment status, anticipated housing upon release, anticipated needs upon release, and study intervention condition. We Fisher’s Exact Tests for categorical measures and used Wilcoxon rank-sum tests for continuous measures. We used a p-value of 0.1 to determine statistical significance due to the small sample size. Data are not available for one qualitative participant who completed a pre-release interview, but was withdrawn from the study prior to release.

## Results

### Participants

We conducted 37 interviews (Figure 1): 24 participants completed pre-release qualitative interviews; of those, 13 also participated in post-release interviews (three of which were with inmates who had been reincarcerated in NCDPS). Most of the participants were heterosexual, middle-aged, single, African American (AA) men and women with limited formal education (Table 1). Substance use before incarceration was pervasive. Most participants were taking antiretroviral treatment before release and many anticipated needing help with getting their medications and health benefits upon release, but did not anticipate needing help with adherence. Substudy participants were more likely to anticipate living with family or friends upon release (p = 0.048), and to report needing help with transportation (p = 0.078) or income assistance (p = 0.020) than other participants in the parent study. Participants who completed both the pre- and post-release qualitative interviews tended to be older (p = 0.027) than those that completed the pre-release interview only.

**Table 1:** **Participant baseline characteristics**

	Cohort comparison: cohort participants not participating in qualitative substudy versus qualitative subset			Qualitative cohort comparison: pre-release only versus both pre- and post-release interviews		
	Non-qualitative cohort	Qualitative subset		Pre-release interviews only	Both pre- and post- interviews	
	(n = 81)	(n = 23) ^a^		(n = 10) ^a^	(n = 13)	
	n (%)	n (%)		n (%)	n (%)	
Variable	Median (IQR)	Median (IQR)	p-value	Median (IQR)	Median (IQR)	p-value
***Demographics***						
Age (years)	40 (33–44)^b^	42 (37–45)	0.318	37 (31–42)	44 (39–46)	0.027*
Black race	63 (78%)	20 (87%)	0.395	8 (80%)	12 (92%)	0.560
Married	11 (14%)	1 (4%)	0.293	1 (10%)	0 (0%)	0.435
Male gender	60 (74%)	16 (70%)	0.790	7 (70%)	9 (69%)	1.000
Less than high school education	32 (40%)^c^	10 (43%)	0.812	3 (30%)	6 (46%)	0.401
Heterosexual orientation	66 (81%)	19 (83%)	1.000	7 (70%)	12 (92%)	0.281
***Incarceration history***						
Number of previous Incarcerations	3 (1–4)^c^	4 (2–5)	0.175	3 (2–4)	4 (2–6)	0.197
Months served for current sentence at baseline	9 (4–23)	10 (2–19)	0.473	10 (7–14)	4 (2–44)	0.950
***Substance use behaviors (3 months prior to incarceration)***						
Alcohol use						
Frequent drinker (2–7 days/week)	43 (54%)^b^	10 (48%)^b^	0.629	3 (37%)^b^	7 (54%)	0.659
Frequent binge (≥5 drinks 2–7 days/week)	36 (46%)^b^	9 (43%)^b^	1.000	2 (25%)^b^	7 (54%)	0.367
Illicit non-injection drug use						
Any	58 (73%)^b^	18 (86%)^b^	0.061	7 (87%)^b^	11 (85%)	1.000
Cocaine	27 (34%) ^b^	9 (43%)^b^	0.457	4 (50%)^b^	5 (38%)	0.673
Crack	39 (49%) ^b^	14 (67%)^b^	0.219	6 (75%)^b^	8 (61%)	0.656
Injection drug use	7 (9%)^d^	1 (5%)^b^	1.000	0 (0%)^b^	1 (8%)	1.000
***HIV diagnosis and treatment status***						
Diagnosed with HIV during current incarceration	22 (28%)^b^	3 (13%)	0.178	0 (0%)	3 (23%)	0.229
Years since HIV diagnosis	5 (2–13)^e^	8 (5–13)^c^	0.324	7 (5–11)	9 (3–13)	0.574
Taking antiretroviral therapy	56 (88%)^g^	18 (86%)^b^	1.000	6 (75%)^b^	12 (92%)	0.531
CD4 Count	337 (183–573)^c^	397 (147–55)6	0.862	374 (158–532)	397 (147–580)	0.078
Viral <400 copies/ml	38 (48%)^c^	12 (52%)	0.812	4 (40%)	8 (62%)	0.414
Post-release HIV care provider identified	50 (62%)	19 (83%)	0.081	8 (80%)	11 (85%)	1.000
***Anticipated housing upon release***						
Living with friends or family	35 (52%)^f^	18 (78%)	**0.048**	9 (90%)	9 (69%)	0.339
Homeless/transitional housing^h^	17 (25%)^f^	5 (22%)	1.000	1 (10%)	4 (31%)	0.340
***Anticipated needs upon release***						
Securing health benefits (e.g., medicaid)	72 (95%)^e^	23 (100%)	0.570	10 (100%)	13 (100)	1.000
Finding a doctor	48 (63%)^e^	13 (56%)	0.628	6 (60%)	7 (54%)	1.000
Getting medications	64 (84%)^e^	22 (96%)	0.628	10 (100%)	21 (92%)	1.000
Adherence to medications	15 (20%)^e^	3 (13%)	0.554	1 (10%)	2 (15%)	1.000
Transportation to clinic or job	46 (61%)^e^	19 (83%)	0.078	7 (70%)	12 (92%)	0.281
Drug addiction treatment	27 (35%)^e^	12 (52%)	0.223	4 (40%)	8 (61%)	0.414
Alcohol treatment	16 (21%)^e^	8 (35%)	0.265	3 (30%)	5 (38%)	1.000
Finding a place to live	51 (67%)^e^	16 (70%)	1.000	8 (80%)	8 (61%)	0.405
Income assistance	61 (81%)^e^	22 (100%)^c^	**0.020**	9 (100%)^c^	13 (100%)	1.000
Getting a job	45 (59%)^e^	16 (70%)	0.467	8 (80%)	8 (61%)	0.405
Mental health services	26 (34%)^e^	8 (35%)	1.000	2 (20%)	6 (46%)	0.379
***Randomized to study intervention condition***	39 (48%)	13 (56%)	0.637	5 (50%)	8 (61%)	0.685

^a^1 participant was withdrawn from study prior to collection of baseline data. ^b^2 missing. ^c^1 missing. ^d^3 missing. ^e^5 missing. ^f^14 missing. ^g^17 missing. ^h^Defined as living in a shelter, street, halfway house, single room occupancy, welfare hotel, or unknown housing arrangements.

*p-value <0.05 indication in bold.

### Qualitative findings overview and theoretical framework: Social Cognitive Theory and HIV Stigma Framework

**Figure 2:**
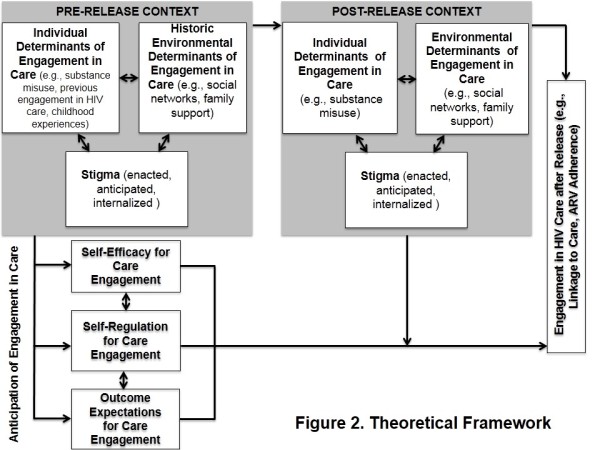
**Theoretical framework.**

The theoretical framework that emerged from the qualitative data (Figure 2) aligns with a conceptual model that integrates the SCT and HSF. Both pre- and post-release, participants discussed specific *individual determinants* (i.e., substance use disorder, feelings about seeking HIV care) and *environmental determinants* (e.g., social networks and neighborhood characteristics) related to their engagement in HIV care, the interplay between themselves and their social and built environments (*reciprocal determinism*), and the effects that each of these had on their engagement in HIV care and health more generally. In addition, during the pre-release interviews participants described beliefs about the importance of engaging in HIV care following release (*outcome expectations),* beliefs around their ability to engage in HIV care and to adhere to ARVs (*self-efficacy*), and *self-regulation* strategies needed to ensure engagement in HIV care and medication adherence (e.g., *goal-setting, enlisting of social support, and self-monitoring*). Furthermore, participants described experiences with HIV-related stigma that reflect *individual attributes* (i.e. *internalized stigma*), *environments* (i.e. *enacted stigma*) and *outcome expectations* (i.e. *anticipated stigma*) that influenced their conscious and subconscious decision-making about engagement in HIV care. We present the salient themes relating to engagement in HIV care that arose from the interviews pre- and post-release with representative quotes illustrating each overarching construct, starting first with those related to individual and environmental determinants, followed by themes related to anticipated engagement in HIV care, and conclude with experiences of stigma. These domains, comparisons of pre- and post-release themes, and relevant theoretical constructs are outlined in Table 2. For the purposes of this manuscript, engagement in care encompasses behaviors identified by participants as important components of their HIV-related medical care, most notably, finding a doctor, attending medical appointments, securing medical and prescription benefits, and adherence to ARVs.

**Table 2:** **Major themes, comparisons pre- and post-release, and relevant theoretical constructs**

	Major themes		
Domain	Pre-release	Post-release	Theoretical construct
***Individual and environmental determinants***	● Substance misuse was viewed as a pervasive, negative influence which led to ART non-adherence, poor health, fractured family networks, and incarceration.	● Substance misuse continued to be a pervasive, negative influence which led to non-adherence, poor health, and fractured social networks, and incarceration.	● SCT, Individual Determinants, Environmental Determinants, Reciprocal Determinism
	● Participants were keenly aware of risk of substance misuse relapse and its association with poor HIV management, but unsure how to avoid relapse.	● Participants were keenly aware of risk of substance misuse relapse and its association with poor HIV management, but many were unable to avoid relapse.	● SCT, Self-Regulation/Self-Monitoring
		● Challenges with housing, stressful life circumstances, and meeting daily needs led to relapse.	● SCT, Reciprocal Determinism
		● Substance use led to ART non-adherence, poor health, and criminal behavior.	● SCT, Reciprocal Determinism
	● Participants desired to avoid past peer networks and to develop new, positive social networks.	● Participants varied in their abilities to develop positive social networks and to reconnect with family members.	● SCT, Self-Regulation/Enlistment of Social Support
	● Peer networks were associated with substance use and criminal behavior.	● Participants who reconnected with past peer networks relapsed and returned to criminal behaviors.	● SCT, Reciprocal Determinism
	● Family networks were strained due to substance use, criminal behavior, or HIV status.	● Participants who developed new social networks (e.g., church groups) or reconnected with family avoided substance misuse.	● SCT, Reciprocal Determinism, Self-Regulation/Enlistment of Social Support
		● Social networks had unanticipated positive role in HIV management (e.g., transportation to appointments, medication reminders, help completing paperwork and securing benefits.	● SCT, Reciprocal Determinism, Self-Regulation/Enlistment of Social Support
***HIV management***	● Participants viewed HIV management as an important part of staying healthy and motivation to avoid substance misuse.	● Participants viewed HIV management as an important part of staying healthy, but HIV care was often eclipsed by substance misuse.	● SCT, Outcome Expectations
	● Participants expressed confidence in their ability to adhere to ART and to manage their HIV.	● Many participants described periods of ART non-adherence and poor health, largely due to substance misuse relapse.	● SCT, Self-Efficacy
	● Participants who had been diagnosed with HIV during previous incarcerations felt they could easily find a doctor, but were worried about paying for medications if they could not find a job.	● Challenges finding employment and meeting basic needs were greater than anticipated. Many participants reported unforeseen challenges in completing paperwork and securing benefits.	● SCT, Outcome Expectations, Reciprocal Determinism
		● Social networks had unanticipated positive role in HIV management (e.g., transportation to appointments, medication reminders, help completing paperwork and securing benefits.	● SCT, Reciprocal Determinism, Self-Regulation/Enlistment of Social Support
	● Newly diagnosed participants were hesitant to access medical care following release due to fears of HIV disclosure.	● Participants continued to be concerned about disclosure, some accessed HIV-related medical care, others did not.	● SCT, Outcome Expectations HSF, Anticipated Stigma
***Stigma***	● Participants recounted numerous counts of experienced HIV-related stigma including losing jobs, rejection by family and friends, public disclosure and harassment which occurred both prior to and during their current incarceration.	● Participants recounted numerous counts of experienced HIV-related stigma and additional stigma associated with criminal history, including losing jobs, rejection by family and friends, public disclosure, and harassment.	● HSF, Enacted Stigma
	● Participants rejected HIV-related services due to fears of HIV disclosure.	● Participants continued to avoid HIV-related services due to fears of HIV disclosure.	● HSF, Anticipated Stigma SCT, Outcome Expectations
	● Participants expressed shame and embarrassment about their HIV status.	● Participants expressed shame and embarrassment about their HIV status and/or their substance misuse relapse.	● HSF, Internalized Stigma SCT, Outcome Expectations

### Individual and environmental determinants of engagement in HIV care

Both before and after release, participants described the historic and present day individual- and environment-level contexts of their lives, and ruminated on the roles these contexts played in shaping their engagement with HIV care both before and after this incarceration.

### Pre-release perspectives

Substance use was the most salient individual-level determinant associated with engagement in HIV care. Every participant viewed substance use as a pervasive influence that was closely linked to the breakdown of their family relationships, experiences of violence, and ultimately their exposure to and difficulties managing their HIV. As one woman stated: “*when I relapse I don’t take my medicine. And that’s not good. The last time I relapsed I didn’t take my medicines.*” *[-AA Female in her 30s]*. Although participants self-monitored the relationship between their substance use, engagement in HIV care, and health, many expressed low self-efficacy in their abilities to prevent their substance use relapse following release. For example, one participant acknowledged her relapse patterns but was unsure how to avert them, saying:

*Every time I get out I do good until like three or four months, and then… I start to relapse. I’m thinking, I don’t know what’s causing me to want to go and do those [drugs]…It’s scary. I don’t know how I am going to handle it, you know, when a problem comes my way. If I’m going to turn to drugs- you know, how I’m going to handle it. -AA Female in her 30s.*

Participants perceived the environments to which they were returning to be rife with obstacles to good health**,** as exemplified by one participant:

*I have to go back to [father’s]…he’s still doing drugs. You know, I know it is not a very safe environment…. I’m scared about it you know… because I don’t want to go back to drugs. That’s what I don’t want. Right now I am doing good on my situation, my sickness [HIV]…I ain’t trying to be dying and it could influence my mind about the drugs, going back to the drugs. –Latino Male in his 30s.*

Most participants described chaotic pre-incarceration environments and fractured family relationships to which they often responded by engaging in violent and illegal activities, including substance use. One man reflected that his mother’s absence during his youth resulted in his approach to solving challenging situations as an adult, noting: “*I ended up… doing a lot fighting [as a child], getting in a lot of violence all the time and so that violence carried on. That’s how I deal with things now when I am threatened with something”. [-AA male in his 50s]*. Notably, many participants’ pre-incarceration social networks revolved around substance use. Relationships with members of networks they perceived to be more wholesome, such as non-using family, were often strained (due to substance use and/or their incarceration), diminishing some participants’ perceived access to relationships that would support them in avoiding relapse following prison release, as exemplified here: *I had a good relationship with my family…they told me in the beginning, you know: ‘if I got into that there [drugs, illegal activities], don’t depend on no help from them and stuff.’ And I chose the wrong ways”. [-AA Male in his 40s]* Some felt that it was “too late” due to severed bonds or a family member’s death during the participant’s incarceration while others were more hopeful and actively working to repair such relationships.

### Post-release perspectives

The post-release interviews revealed complex interactions between individual factors (e.g., substance use) and post-release social and built environments. As participants anticipated, following release, substance use presented an acute challenge for most and hence emerged as a salient theme. Those who did relapse associated their drug use with nonadherence and poor health (e.g., weight loss) as illustrated by one participant, who stated: “*Man, in the streets if you’re out there on drugs [illicit] and stuff like that there, you might [not] take your medicine. You might [not] eat.*” *[-AA Male in his 40’s]*. Substance use was often precipitated by both individual-level and environmental determinants, including associations with former substance using networks, stressful life events, or survival needs, as described by one man:

*You know, I came home and I needed some things…I tried to go get a voucher…I couldn’t get any…So, I met some friends…I said ‘I need y’alls help…They were dopers…They know I sell dope. They knew I knew dope. That’s what I knew. I sell it. I sell that dope, that’s what I do. -AA male in his 40s.*

Participants’ experiences with substance use and ultimately, their engagement in HIV care**,** were intertwined with the post-release environment, particularly housing situations. Homelessness, and housing insecurity more generally, was a predominant theme throughout the post-release interviews. Participants avoided shelters when possible, which they feared would connect them with negative peer influences, and described prolonged periods of transition, such as moving between friends and family, or staying in single room occupancy (SRO) hotels for as many nights as they could afford before returning to the streets. Participants saw a direct connection between the safety of their neighborhood environment, substance use relapse, and their engagement in HIV care. As one man described:

*I really didn’t have anything…I stay[ed] in an abandoned building or under the bridge or even an abandoned car…You know, sleeping in areas and stuff like that, you never know what might happen. You doze off to sleep, there’s no telling who might come by…And so to try to ease to those thoughts…I usually would pick up, use. -AA Male in his 40’s.*

While this participant went on to describe stopping his ARVs as a result of this relapse, he also discussed that when he entered a long-term residential substance use treatment program which provided structured programming, long-term housing, and facilitated formation of new social networks, his cravings to use diminished and his medication adherence improved.

As in pre-release interviews, participants described both the positive and negative role of social networks following release. Participants who had reestablished relationships with non-substance using family members or developed new networks through Narcotics Anonymous (NA) or religious groups found the support they needed to avoid negative influences**,** noting:

*And, as I say, I’ve got support with my church, my family, you know, NA groups…It was a struggle at first… I couldn’t have did it without my support systems that I’ve got, my family and my church…So, I had to avoid my old friends and get some new friends. And that was kind of hard because I was so used to those old friends. -AA female in her 30s.*

Most participants who experienced substance use relapse, and all who were reincarcerated post-release, attributed these negative outcomes to their substance using social networks. Although these participants expressed pre-release the need to connect with “positive-thinking people”, once released, they discussed being unable to engage with new networks. As this participant related:

*Well, the tipping point was, OK, I was home for like 60 days trying my best not to do nothing ‘cause I was glad to be out…Then I got around those guys again. That was it. When I got around those guys again, into the same playground, the same playmates….I never really ever thought about ‘how do I find positive people.’ I don't know. I’m so used to being around negative people, you know. -AA male in his 40s.*

A number of participants either did not have relationships with family members or did not disclose their HIV status to family members following release. However, although not anticipated pre-release, following release, participants who had disclosed their HIV status to supportive family or friends noted that these relationships provided important support for linkage to care and medication adherence. One woman described how family members reminded her to take her ARVs, saying: “*He’ll hand me my medicine and he’ll get me a bottle of water and then he’ll say ‘I know you’ve got to have this.’” [-AA Female in her 40s]*. Several participants, particularly those with low literacy, described how family members had helped them get to appointments and navigate paperwork associated with reinitiating HIV-related benefits as illustrated by this quote:

*[I] went to a couple clinics. And me and my niece, we signed a couple papers…She went to college. She’s very smart…I can read and write a little bit, so I can go to her and say ‘well, I’m going to see these people and I need to sign these papers and they help to get my medication. Help me fill out the application…I need health care.’ She got it for me. -AA Male in his 40s.*

While such family support helped, unanticipated administrative barriers (e.g., time required to process paperwork) often hindered timely linkage to care; these barriers were particularly salient for low literacy participants who had limited or no outside help navigating administrative systems. Even those who successfully accessed HIV services faced significant challenges to securing employment and housing, and as a result, their struggles to meet basic needs hindered health care access.

### Anticipated and actual outcomes: engagement in HIV care

Emergent themes about engagement in HIV care, described below, revealed a complex juxtaposition between what participants anticipated their engagement in HIV care would be like when they reentered their community and their actual behaviors and engagement in HIV care following release.

### Pre-release perspectives

During the pre-release interviews, participants expressed high self-efficacy for medication adherence and positive outcome expectations for engagement in HIV care. They saw themselves readily attending HIV-related medical care appointments, taking medication, and maintaining their health more generally (e.g., eating well) as would be needed to avoid becoming too sick to work or care for themselves or others, or even dying. Many believed their HIV diagnosis motivated them to avoid drugs and alcohol, as illustrated here:

*Someone who is not positive, they wouldn’t think too much about needing to stay healthy and exercising. They wouldn’t take it as seriously. They wouldn’t take drugs and alcohol, staying off that, as seriously. It wouldn’t be a real death blow to them as it is me. -AA Man in his 40s.*

Participants diagnosed before this incarceration felt confident they would be able to find an HIV care provider upon release, but worried about how to pay for their medications if they could not secure a job or benefits. In contrast, people diagnosed during this incarceration expressed low self-efficacy for finding a doctor and initiating HIV care after release. For these participants, their confidence in their ability to initiate HIV care was undermined by concerns that accessing such care could disclose their serostatus to others (e.g., anticipated stigma). As one man exemplified:

*[W]hat am I going to do when I get out? Do I have to go see the doctor, do I have to go see the medical people? What will people think about me? What will people do?…I might be in a room and pick up my meds and people say ‘Hey, hey. What you doing up here?’ -AA Man in his 30s.*

### Post-release perspectives

Following release, participants continued to emphasize the importance of linking to HIV care and medication adherence as part of maintaining their overall health. As one participant described: *“As long as I take the medicine and I do what I’m supposed to do, I live, I’m living a normal life. Blood pressure, excellent. Pulse, excellent. Respiration, excellent. All that stuff is in good shape. No problem!” [-AA Man in his 40s]*. However, participants’ ability to successfully attend medical appointments and adhere to ARVs was ultimately intertwined with their substance use; which was in turn a function of social interactions, including experiences of stigma and stressful life events. As noted previously, several participants described being nonadherent to medications when experiencing short or prolonged periods of substance use relapse. Despite their keen awareness of and concerns about the impact of nonadherence on their health, taking their ARVs was eclipsed by their addiction. As one participant explained:

*When I was on the street I was drinking and drinking. But back in my mind I was thinking ‘damn, you’re speeding up the process! You’ll be in a casket real soon.’ But I’m so addicted…I tried that [taking ARVs] for a while. But it makes you feel so nauseated. It makes it very hard. -AA Man in his 30s.*

### Experiences with HIV-related stigma

Experiences with stigma emerged as a cross-cutting, multilevel theme. Every participant described experiences of HIV-related stigma, including enacted stigma, anticipated stigma, and internalized stigma both pre- and post-release [32].

### Pre-release perspectives

Participants described numerous episodes of experiencing enacted stigma prior to and while incarcerated. These included being fired from jobs as a result of HIV disclosure; rejection by family, friends and community members; and public disclosure and harassment on the prison yard. Memories of these experiences were so painful that some participants felt unable to return to specific neighborhoods. The deleterious effect of experiences of enacted stigma on participants’ well-being are exemplified by this man:

*I came back to the yard [from the prison infectious disease clinic] and everyone was saying…that I had HIV… I was so upset, I went and to the individual that was talking to the yard about my medical condition…sometimes people are naïve to AIDS, you know, and it just got all over the yard…I was quite upset about it…There was one guy on the yard that was saying it and he has the same thing that I have. I know he does, ok. Alright. I’m not dumb ok, but I didn’t take his business to the yard. I didn’t, but in return he took my business to the yard…I felt stabbed in the back. -AA Male in his 40s.*

Some participants displayed anticipated stigma: they self-isolated and hesitated to accept available HIV-related services in order to avoid HIV disclosure and its potential stigma. One woman, who had been diagnosed during her current prison sentence, chose uncertain housing over arranging to go to a halfway house for people with HIV after release:

*A halfway house is definitely out because, like I said, I’ve been in recovery before and all the halfway houses in the city…they’re known to be boarding them with AIDS and, you know, the stigma. You know, I mean ‘That’s that woman with HIV. That’s the house where they have AIDS’…I want to be known for who I am and not what I have, you know? It’s a difficult time. -AA Female in her 40s.*

Participants also experienced internalized stigma, noting that they “tried not to think about it [HIV]” or felt shame around their HIV status, as one woman described: “*I don’t want nobody to know nothing, nothing about this because I am embarrassed…I don’t want people to turn away from me and stuff thinking that I have something that is contagious.” [-AA Female in her 40s]*.

### Post-release perspectives

Following release, participants also described a range of experiences with HIV-related stigma, the majority of which were enacted. One participant was fired because he was seen entering a local AIDS Service Organization (ASO):

*I ended up getting a job and then someone seen me going around [ASO] and then it got back to my job. And then it was an HIV thing…And I wasn’t trying to hide it… But the man who owns, he had a real problem with it…I’m not welcome around that store anymore. -Native American Male in his 30s.*

Participants experienced additional layers of enacted and internalized stigma due to other stigmatizing conditions (e.g., incarceration, substance use) that they described negatively affecting their ability to prioritize HIV care including: profiling by law enforcement, rejection from jobs, denial of housing or benefits, public harassment, and shame surrounding substance use. One man avoided seeking HIV care because he felt ashamed of his appearance when he relapsed, saying: “*Lots of programs were there, you know, agencies and stuff like that but I wasn’t coming out into the public. You know, when I’m [using], I look, my appearance and stuff like that, nah, I wasn’t coming out.” [-AA man in his 40s]*. Another man described being singled out during a job training program because of his criminal record: “*One guy’s wife in the program…she picked on me. ‘I don’t know who gonna hire you.’ She was very discouraging to me.” [-AA Man in his 50s]*. The same participant described how this experience, combined with the challenging training, lowered his self-esteem, led him to reconnect with substance using networks, and to ultimately relapse and stop taking his HIV medications. Participants had varying experiences related to stigma from family and friends following release from prison. One participant who, before release, had described how her father, upon learning her serostatus, had kicked her out of their house, talked, after release, about how her sobriety helped her and her father rebuild their relationship.

## Discussion

These findings underscore the competing demands weighing on many released inmates, and highlight how engagement in HIV care, while valued as important by most HIV-infected prisoners, may become eclipsed by more immediate concerns (e.g., addiction, homelessness). Participant descriptions of the release process and their lives post-release overwhelmingly highlighted numerous acute challenges, including the difficulties managing their personal circumstances and environmental pressures while avoiding substance use relapse and criminal activity. When viewed within the framework of SCT and the HSF, these results illuminate the contrast between the anticipated release environment and the even more difficult realities of this tumultuous period, and the role of self-efficacy and regulatory behaviors in navigating these challenges. This study adds to our understanding of engagement in HIV care among recently released HIV-infected prison inmates in the following key ways: 1) It is one of the first theory-guided studies to qualitatively explore relationships between pre-release and post-release expectations for engagement in HIV care following release from prison; and 2) is among the first studies to identify how experiences of compounded stigma influence engagement in HIV care following release from prison.

For HIV-infected inmates, linkage to HIV care and treatment cannot be implemented without an acknowledgement of the role that substance use plays in effective HIV management [43–45]. Viewed within the context of the SCT, substance use disorders and cycles of substance use relapse, treatment, and remission colored every aspect of the individual’s interaction with his or her environment and ultimately affected adherence and accessing services. Substance use among individuals with a history of incarceration in jail and prison is common [25, 44, 46]. In this study, relapse was triggered by stressful life situations, such as homelessness, reintegration with substance using networks, experiences of enacted stigma, and economic pressures. Traditional safety nets, such as homeless shelters, may be destabilizing through exposure to negative peer networks. Active alcohol and other drug use has been associated with medication nonadherence, including among individuals entering jail and the homeless [46–49]; this finding was echoed in the qualitative interviews. However, treatment of substance use disorders is associated with medication adherence and engagement in care; treatment may allow people who use drugs to achieve parity in HIV treatment outcomes, as compared to people who do not use drugs [50]. Holistic programming that incorporates the role that past experiences, such as childhood trauma, play in current life choices, while also focusing on evidence-based approaches for the treatment of substance use disorders (including medication-assisted therapies), individual skill and resource development (e.g., securing housing, identifying non-using social networks) may reduce substance use relapse [51–53]. Our findings underscore the need for such programming, particularly among HIV-infected inmates. Despite the evidence supporting substance use disorder treatment as a means to improve engagement in HIV care, a small percentage of releasees nationally are linked to substance abuse treatment either in prison or upon release, and in-prison substance use disorder treatment programs are curtailed by a lack of funding and the relatively short sentences of some offenders [54]. Treatment may be further complicated by the lack of medication-assisted therapies for people who use cocaine or crack. Crack and cocaine use was reported by the majority of substudy participants prior to incarceration (67% and 43% respectively); other studies have found prevalent use of crack/cocaine in HIV-infected individuals entering and leaving jails [44, 46].

Consonant with past research, participants viewed involvement in positive social networks as playing an important role in avoiding substance use and criminal activity [43, 55–57]. This was particularly relevant for participants who received instrumental and emotional support with respect to their engagement in HIV care. Social support systems have been associated with positive HIV-related outcomes, as reflected in our qualitative data [49, 58], however, many participants struggled to identify new social networks upon release. Stigma and other factors related to incarceration may inhibit family-based social network supports for many HIV-infected inmates, highlighting the importance of enlisting social networks outside of family (e.g., church, NA). Employment-based programs offer the potential to integrate skills development while helping former inmates to learn to navigate “real world” contexts, build new social networks, and demonstrate self-efficacy [59]. Evaluation of vocational programming for ex-offenders has been hampered by a lack of intervention programming with a strong research design [60, 61]. In response, several national level job development programs are currently being implemented and evaluated, although the vast majority focus only on men [62].

Over 80% of participants were on ART prior to release and almost all participants reported needing help securing medical benefits (e.g., Medicaid) or getting medications upon release. Although at pre-release participants expressed high self-efficacy for linking to care and adherence to HIV medications after release, prior analyses of this cohort revealed that roughly half of participants had not accessed medical care within four weeks of release, at which time ARVs provided by the NCDPS upon release would be exhausted [37]. Similarly, other studies have found low linkage to HIV care among HIV-infected jail and prison releasees [13, 24, 63, 64]. These findings suggest that confidence (self-efficacy) may not translate to engagement in HIV care following release, particularly if lived experiences differ from outcome expectations. Inmates may benefit from programming that addresses outcome expectations for engagement in HIV care upon release, potential barriers to meeting these expectations (e.g., social interactions, substance use relapse), and the concrete skills and behaviors (self-regulation strategies) required to engage in care when faced with the realities of life upon release. For example, releasees may not have sufficient health literacy skills, photo identification, phone access to navigate the process of reestablishing care, or underestimate the paperwork burden required to do so. Past research suggests that HIV-infected inmates who receive help completing paperwork following release are more likely to fill ARV prescriptions [13].

Limited research has explored the role of stigma on HIV-related outcomes for former HIV-infected prison inmates [21, 26–28]. Notably, although the qualitative interview guide used for this study did not include questions or probes specifically related to stigma, participants described countless experiences with stigma that both directly and indirectly affected their ability to successfully engage in HIV care and treatment. These findings suggest that low uptake of HIV care by HIV-infected inmates following release from prison may in part stem from fear of stigma, either due to past experiences or internalized feelings. Similarly, stigma may also prevent HIV-infected inmates who are not eligible for traditional safety net benefits due to their criminal history from accessing needed support through AIDS-specific resources (e.g., housing). Roughly 25% of participants anticipated being homeless upon release. Homelessness or other transitory living situations may make it more difficult to establish and maintain care [14, 64] and has been associated with increased sexual risk, substance use, and poor HIV-related outcomes [16, 48, 65–67]. A richer understanding of the role of stigma, particularly the intersectionality of non-HIV-associated and HIV-associated stigmas that HIV-infected prisoners experience, on barriers to linkage and retention in care is needed to inform the design of future programming for HIV-infected releases.

This study is one of the first to explore HIV-infected inmates’ pre-release outcome expectations for engagement in HIV care post-release and how these perceptions compared with actual experiences post-release. This is a preliminary step in designing programs that encompass the transition from prison to the community, and bridge the gap between HIV-infected inmates’ needs (both perceived and real) and their ability to engage in HIV care throughout this transition. Study findings should be interpreted in light of the small sample size, which was collected using convenience sampling. However, past research has indicated that eight interviews are sufficient for saturation [68] and the qualitative subsample did not differ significantly from the overall cohort for the majority of baseline measures. In addition, participants received either pre-release discharge planning (standard of care for the state of NC) or intensive case management (study intervention). It is possible that participants were more acutely aware of their anticipated needs following release or had more of these needs met following release as a result of discharge planning or case management. We are unable to speak to the release experiences of participants that did not complete the post-release interview, but based on baseline data, those who and did not follow-up had few discernable differences in regards to their demographic characteristics and self-reported needs.

## Conclusion

Given high recidivism rates among incarcerated populations in general and poor HIV-related outcomes for HIV-infected inmates after release, a fundamental disconnect exists between an individual’s expectations of life and the actual outcome after release. This disconnect may be due to the difficulty of exerting self-influence over the competing pressures of the reentry environment and changing personal circumstances. Taken together, these findings highlight the challenges HIV-infected inmates face upon release and the need for multicomponent HIV linkage and care programming which extends pre- and post-release and addresses: 1) evidence-based substance use disorder treatment; 2) stigma; 3) environmental factors that precipitate relapse (e.g., homelessness); 3) outcome expectation management between pre-release expectations and the realities of release, and; 4) practical problem-solving and skill development. In addition, there is need for future research that explores the conscious and subconscious effects of layered stigma on engagement in HIV care among HIV-infected inmates and ways to minimize the corrosive effects of stigma on successful community reentry and well-being. The attainment of these goals has the potential to enhance not only the well-being of HIV-infected inmates, but that of the communities to which they return.

## Funding

This study received grant support from the National Institute of Mental Health, National Institutes of Health (R01 MH068719) and the University of North Carolina at Chapel Hill’s Center for AIDS Research (AI 50410–04). DFH’s time was supported by the National Institute of Mental Health of the National Institutes of Health under Award Number F31MH105238, the George W. Woodruff Fellowship of the Laney Graduate School, Emory University, and the UNC-Chapel Hill IMPACT Award. CEG’s time was partly supported by K24 HD069204-01A1. CEF’s time was partly supported by F32 DA030268-01. DLR’s time was partly supported by R21 MH099162-01A1.
